# Cognitive function and its transitions in predicting all-cause mortality among urban community-dwelling older adults

**DOI:** 10.1186/s12888-020-02618-9

**Published:** 2020-05-06

**Authors:** Mu-Cyun Wang, Tsai-Chung Li, Chia-Ing Li, Chiu-Shong Liu, Chih-Hsueh Lin, Wen-Yuan Lin, Chuan-Wei Yang, Shing-Yu Yang, Cheng-Chieh Lin

**Affiliations:** 1 Department of Family Medicine, China Medical University Hospital, Taichung, Taiwan; 2 School of Medicine, College of Medicine, China Medical University, Taichung, Taiwan; 3 Department of Public Health, College of Public Health, China Medical University, Taichung, Taiwan; 4 Department of Medical Research, China Medical University Hospital, Taichung, Taiwan; 5 Department of Healthcare Administration, College of Medical and Health Science, Asia University, Taichung, Taiwan

**Keywords:** Cognitive function, Cognitive transition, Mini-mental state examination, Mortality

## Abstract

**Background:**

Cognitive impairment is accompanied with high rates of comorbid conditions, leading ultimately to death. Few studies examine the relation between cognitive transition and mortality, especially in Asian population. This study evaluated baseline cognition and cognitive transition in relation to all-cause mortality among community-dwelling older adults.

**Methods:**

We conducted a community-based prospective cohort study among 921 participants of Taichung Community Health Study for Elders in 2009. Cognitive function was evaluated by the Mini-Mental State Examination. Cognitive impairment was considered if the total score is less than 27, 24, and 21 for a participant’s educational level of more than 6 years, equal or less than 6 years, and illiteracy, respectively. One-year transition in cognitive function was obtained among 517 individuals who were assessed in both 2009 and 2010. Mortality was followed up until 2016. Cox proportional hazards models were applied to estimate the adjusted hazard ratios of mortality for baseline cognitive impairment and one-year transition in cognitive status.

**Results:**

After a follow-up of 6.62 years, 160 deaths were recorded. The multivariate adjusted hazard ratio (95% confidence interval) for baseline cognitive impairment was 2.08 (1.43, 3.01). Significantly increased mortality risk was observed for cognitively impaired–normal and impaired–impaired subgroups over 1 year as compared with those who remained normal [2.87 (1.25, 6.56) and 3.79 (1.64, 8.73), respectively]. The area under the receiver operating characteristic curves demonstrated that baseline cognition and one-year cognitive transition had no differential predictive ability for mortality. Besides, there was an interaction of cognitive impairment and frailty, with an additive mortality risk [5.41 (3.14, 9.35)] for the elders who presented with both.

**Conclusion:**

Baseline cognitive impairment rather than one-year progression is associated with mortality in a six-year follow-up on older adults.

## Background

The world’s population is now rapidly aging. The global population aged 65 and above is expected to grow from 506 million in 2008 to 1.4 billion by 2040, with the percentage doubling from 7 to 14% [1]. In Taiwan, the percentage of individuals aged 65 and above was 7% in 1993 and 14% in 2018, showing that the country has become one of the fastest aging countries in the world. Taiwan only spent 25 years to transition from an “aging society” to an “aged society,” indicating that the burden of medical care and support for older adults has become heavier. Cognitive impairment is associated with aging [2, 3]. With an increasingly aged population, cognitive impairment becomes a major challenge for public health.

A nationwide survey based on the 2010 population census of Taiwan showed that the age-adjusted prevalence of dementia (including very mild dementia) and mild cognitive impairment in adults aged 65 and above was 8.04 and 18.76%, respectively [4]. Thus, cognitive impairment is one of the most common health problems of older adults in Taiwan. Cognitive impairment is associated with significant functional dependence [5] and poor quality of life [6]. It is also related with adverse physical, psychosocial, or economic consequences in their families. Cognitive impairment is accompanied with high rates of comorbid conditions [7], resulting in an increased use of home healthcare [8], hospitalization [9], nursing home care [10], and ultimately death.

Previous studies reported that cognitive function [11–16] and its combination with frailty [17–21] are associated with increased risk of mortality. The majority of these studies focused on populations from countries in Europe and America. Studies also reported that cognitive function [22, 23] and cognitive transition [24] predicted mortality in community-dwelling Chinese older adults. Other studies from Asian community-dwelling older adults have been reported, namely, Taiwan [19], Korea [18], Japan [25], and Israel [17]. In the Taiwan study, the association between cognitive frailty and all-cause mortality was reported, where the former was determined by the concomitant presence of dynapenia and cognitive declines in any domains [19]. In the Japan study, declines in global cognitive score or subscale scores were independent predictors of all-cause mortality [25]. In the Korea study, frailty and cognitive impairment independently predicted mortality, and a significant interaction between frailty and cognitive impairment was reported [18]. In the Israel study, frailty was shown to be associated with an increased mortality among the oldest old but cognitive impairment was not [17]. Few studies have explored whether cognitive transition has any prognostic significance with respect to survival among community-dwelling older adults. Therefore, the objectives of this study were to explore the associations of baseline cognitive impairment and cognitive transition during a one-year period with all-cause mortality among participants of the Taichung Community Health Study for Elders (TCHS-E).

## Methods

### Study design and participants

**Fig. 1:**
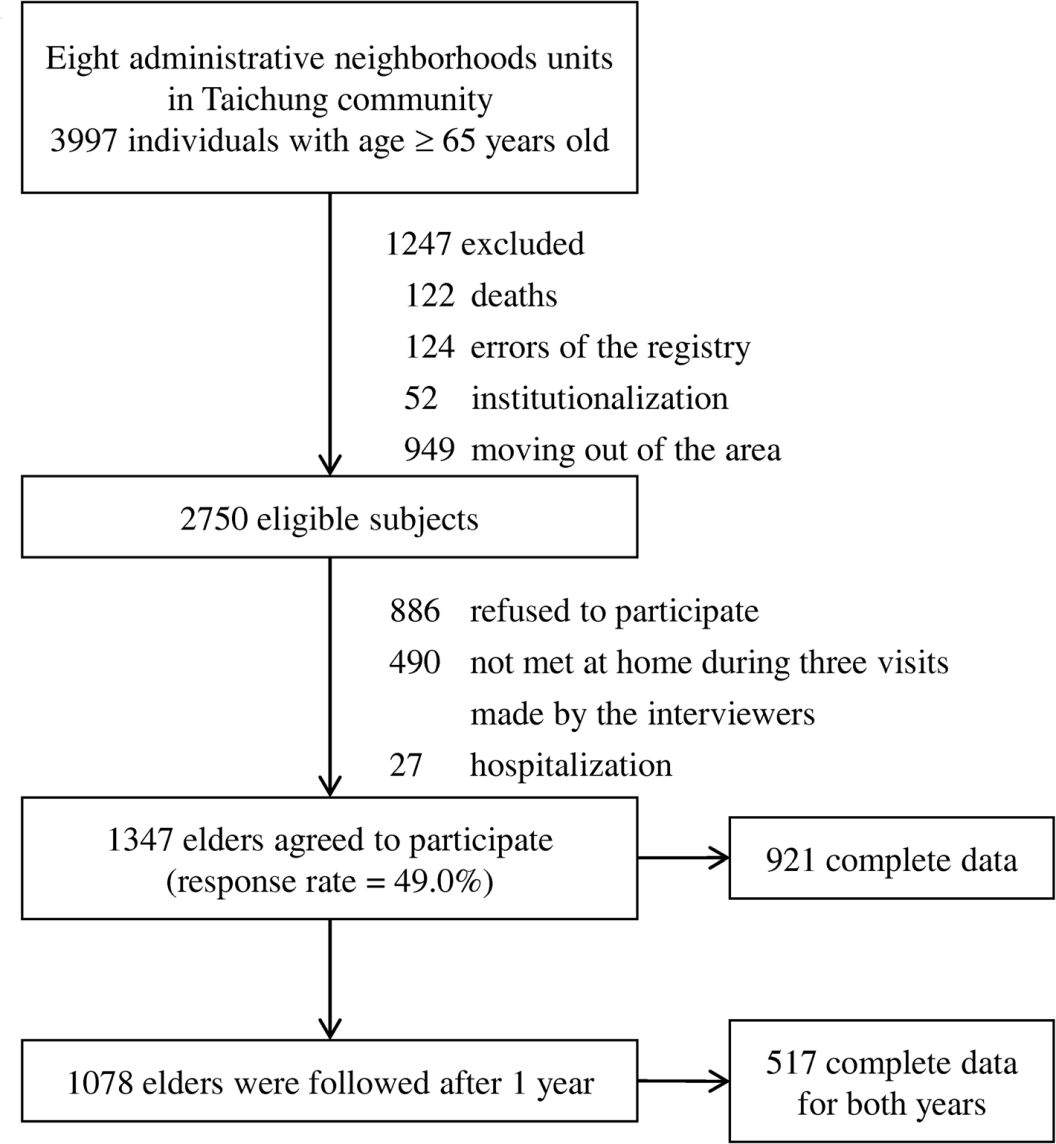
The flowchart of recruitment procedures of the current study

A community-based cohort study was conducted among the participants of the TCHS-E. A total of 3997 residents aged 65 and above in eight administrative neighborhoods of the North District of Taichung City in June 2009 were invited to participate in the study. Through letters, phones, and home visits, we excluded 1247 individuals for the reasons of death, institutionalization, moving out of the area, and errors of the registry. Among the remaining 2750 eligible individuals, 1347 (49.0%) accepted our invitation to participate, and 1078 individuals were reassessed in 2010. The inclusion criteria, exclusion criteria, and reasons for attrition were mentioned in another paper [26]. We used 921 participants with complete data for baseline cognitive status in 2009, and 517 participants with cognitive measurements in both 2009 and 2010 for change in cognitive status. Figure 1 shows the flowchart of recruitment procedures. All participants were followed up until death or to the end of the study. The TCHS-E was approved by the Human Research Committee (HRC) of China Medical University Hospital (DMR 97-IRB-055), with the written informed consent obtained from each participant. The secondary data analysis in the current study was also approved by the HRC (CMUH107-REC1-169).

### Data collection

All participants underwent a face-to-face interview using a standardized questionnaire, which consisted of age, sex, educational level, marital status, smoking, alcoholic drinking, leisure-time physical activity, chronic diseases, fall history, sleep disturbance, and medication, in 2009. The body mass index (BMI) was calculated as the weight in kilograms divided by the square of height in meters (kg/m^2^). Based on the World Health Organization, underweight is defined as BMI < 18.5 kg/m^2^, overweight as 25 ≤ BMI < 30 kg/m^2^, and obesity as BMI ≥ 30 kg/m^2^. Frailty was defined as a clinical syndrome that consists three or more of the five phenotypic components: shrinking, weakness, poor endurance and energy, slowness, and low physical activity level. The criteria for the Cardiovascular Health Study Group proposed by Fried et al. were adopted [27], except shrinking was defined as unintentional weight loss of ≥3 kg in the prior year as an adjustment in proportion to Chinese body build [28]. Further details had been described in our previous work [26].

### Cognitive function measurements

The Mini-Mental State Examination (MMSE) has now become a widely used instrument to screen for cognitive disorders and tract changes in cognitive functioning [29]. The MMSE contains items about orientation to time and place, registration, attention and calculation, memory, language, and visual constructional ability. The total score ranges from 0 to 30, and the cut-point score varies with different educational levels. Trained research assistants administered the MMSE. Cognitive impairment was considered if the total score of MMSE is less than 27, 24, and 21 for a participant’s educational level of more than 6 years, equal or less than 6 years, and illiteracy, respectively. The cut-points were determined by a slight modification from literature in Chinese and Korean versions of MMSE [30, 31].

### Death ascertainment

The primary outcome was all-cause mortality, which was determined by a computer linkage with a unique identification number to the death records from the Health and Welfare Data Science Center database. The length of the follow-up was determined as the interval from the date of recruitment to the date of death or to the end of the study (December 31, 2016).

### Statistical analysis

The baseline characteristics were reported and compared across cognitive status by the chi-squared test for categorical variables and *t* test for continuous variables. Cognitive function was categorized into normal or impaired using a cut-point score for MMSE in accordance with the participant’s education level. One-year transition in cognitive function was then derived by the cognitive status in 2009 and 2010. Cognitive transition was classified into four combinations: remaining normal (normal–normal), converting from normal to impaired (normal–impaired), reverting from impaired to normal (impaired–normal), and remaining impaired (impaired–impaired). The Cox proportional hazards models were fit to estimate the hazard ratios (HRs) and 95% confidence intervals (CIs) of all-cause mortality for continuous baseline MMSE scores, binary baseline cognitive status, and one-year transition in the cognitive status. The proportionality assumption was tested by including an interaction term of each baseline variable and follow-up time. The areas under the receiver operating characteristic curves (AUCs) were calculated to evaluate the ability of baseline cognitive function and one-year transition in cognitive function to correctly classify mortality status. The nonparametric method was used to test differences in their AUCs [32]. The survival functions were estimated by the Kaplan–Meier method, and log-rank tests were used to determine the differences in entire survival functions among groups. The interaction of cognitive impairment and frailty was estimated by stratification at baseline: cognitively normal and nonfrail, cognitively impaired but nonfrail, cognitively normal but frail, and cognitively impaired and frail. All analyses were performed with SAS version 9.4 (SAS, Cary, NC). All *p* values were two-tailed, and a *p* value of < 0.05 was considered statistically significant.

## Results

The baseline socio-demographic factors, lifestyle behaviors, disease history, and frailty status of the 921 participants are shown in Table 1. Those who had cognitive impairments were older, female predominant, less married, and had lower education levels. They also had less alcohol consumption and less leisure-time physical activity. Stroke, cataract, fall, and frailty were more common in cognitively impaired older adults than in cognitively normal ones. For frailty components, the proportions of low physical activity, slowness, and weakness were also higher in the cognitively impaired group than in the control group.

**Table 1:** Comparisons of baseline socio-demographic factors, lifestyle behaviors, disease history, and frailty status according to cognitive status

Variables	Cognitive impairment N (%)		*p* value
	No (*N* = 779)	Yes (*N* = 142)	
***Socio-demographic factors***			
Men	427 (54.81)	51 (35.92)	< 0.001
Age (years)	73.26 ± 6.02	78.15 ± 7.27	< 0.001
65–74	493 (63.29)	47 (33.10)	
75–84	249 (31.96)	63 (44.37)	
≥ 85	37 (4.75)	32 (22.54)	
Education			< 0.001
No education	68 (8.73)	36 (25.35)	
Primary education	217 (27.86)	44 (30.99)	
Secondary or tertiary education	494 (63.41)	62 (43.66)	
Married	582 (74.71)	77 (54.23)	< 0.001
Body mass index (kg/m^2^)	24.43 ± 3.42	23.95 ± 3.82	0.13
< 18.5	26 (3.34)	11 (7.75)	
18.5–24.9	449 (57.64)	80 (56.34)	
25–29.9	264 (33.89)	42 (29.58)	
≥ 30	40 (5.13)	9 (6.34)	
***Lifestyle behaviors***			
Smoking	72 (9.24)	12 (8.45)	0.87
Alcohol drinking	107 (13.74)	10 (7.04)	0.04
Physical activity	576 (73.94)	90 (63.38)	0.01
***Disease history***			
Hypertension	390 (50.06)	81 (57.04)	0.15
Diabetes Mellitus	125 (16.05)	27 (19.01)	0.45
Heart disease	227 (29.14)	51 (35.92)	0.13
Hyperlipidemia	200 (25.67)	30 (21.13)	0.30
Gout	87 (11.17)	16 (11.27)	1.00
Hyperuricemia	76 (9.76)	19 (13.38)	0.25
Arthritis	154 (19.77)	29 (20.42)	0.95
Osteoporosis	136 (17.46)	30 (21.13)	0.35
Stroke	39 (5.01)	17 (11.97)	0.003
Cataract	333 (42.75)	90 (63.38)	< 0.001
Cancer	136 (17.46)	30 (21.13)	0.35
Fall history	160 (20.54)	55 (38.73)	< 0.001
Sleep disturbance	345 (44.29)	71 (50.00)	0.24
***Frailty status***	63 (8.09)	38 (26.76)	< 0.001
***Frailty components***			
Shrinking	98 (12.58)	23 (16.20)	0.30
Poor endurance and energy	28 (3.59)	9 (6.34)	0.19
Low physical activity	132 (16.94)	35 (24.65)	0.04
Slowness	238 (30.55)	93 (65.49)	< 0.001
Weakness	202 (25.93)	72 (50.70)	< 0.001

Mean ± standard deviation for continuous variables; numbers (percentage) for categorical variables

After a mean follow-up of 6.62 years, 160 deaths among the 921 participants were recorded. The overall all-cause mortality rate was 26.26 per 1000 person-years. 108 deaths among the 779 cognitively normal participants and 52 deaths among the 142 cognitively impaired participants were recorded. After multivariate adjustment for comorbidities and frailty, the HR (95% CI) of all-cause mortality for one point increase in MMSE was 0.93 (0.90, 0.97). To dichotomize MMSE scores by the above cut-point scores based on education levels, the multivariate-adjusted HR (95% CI) for baseline cognitive impairment was 2.08 (1.43, 3.01). A total of 517 subjects remained to be assessed in 2010, which were then used to derive changes in the cognitive status. Compared with those remaining cognitively normal over one-year period, impaired–normal and impaired–impaired subgroups were at a higher risk of all-cause mortality, with HR (95% CI) of 2.87 (1.25, 6.56) and 3.79 (1.64, 8.73), respectively. However, the normal-impaired subgroup could not predict mortality. The results are shown in Table 2.

**Table 2:** Comparisons of all-cause mortality according to baseline cognitive status and one-year transition in cognitive status

Variables	N	Deaths	Person- years	Incidence rate	Age and sex -adjusted	Multivariate-adjusted^a^	Multivariate-adjusted^b^
					HR (95% CI)	HR (95% CI)	HR (95% CI)
**Baseline MMSE score**					0.91 (0.88, 0.94)***	0.91 (0.88, 0.94)***	0.93 (0.90, 0.97)***
**Baseline cognitive status**							
Normal	779	108	5244.40	20.59	1.00	1.00	1.00
Impaired	142	52	849.34	61.22	2.60 (1.81, 3.73) ***	2.53 (1.76, 3.65)***	2.08 (1.43, 3.01)***
Total	921	160	6093.74	26.26			
**One-year transition in cognitive status**							
Normal-normal	393	31	2368.37	13.09	1.00	1.00	1.00
Normal-impaired	55	9	315.73	28.51	2.19 (1.02, 4.71)*	2.36 (1.07, 5.19)*	2.01 (0.88, 4.59)
Impaired-normal	31	9	168.36	53.46	3.27 (1.51, 7.09)**	3.60 (1.64, 7.90)**	2.87 (1.25, 6.56)*
Impaired-impaired	38	13	196.41	66.19	4.83 (2.29, 10.15)**	5.26 (2.45, 11.32)***	3.79 (1.64, 8.73)**

*: *p* < 0.05; **: *p* < 0.01; ***: *p* < 0.001

Incidence rate (number of incident cases) / (1000 person-years), *HR* Hazard ratio, *CI* Confidence interval

^a^Multivariate adjustment for age, sex, education, marital status, BMI, smoking, alcohol drinking, physical activity and exercising program

^b^Multivariate adjustment for age, sex, education, marital status, BMI, smoking, alcohol drinking, physical activity, exercising program, hypertension, diabetes mellitus, heart disease, hyperlipidemia, gout, hyperuricemia, arthritis, osteoporosis, stroke, cataract, cancer, fall history, sleep disturbance and frailty

**Fig. 2:**
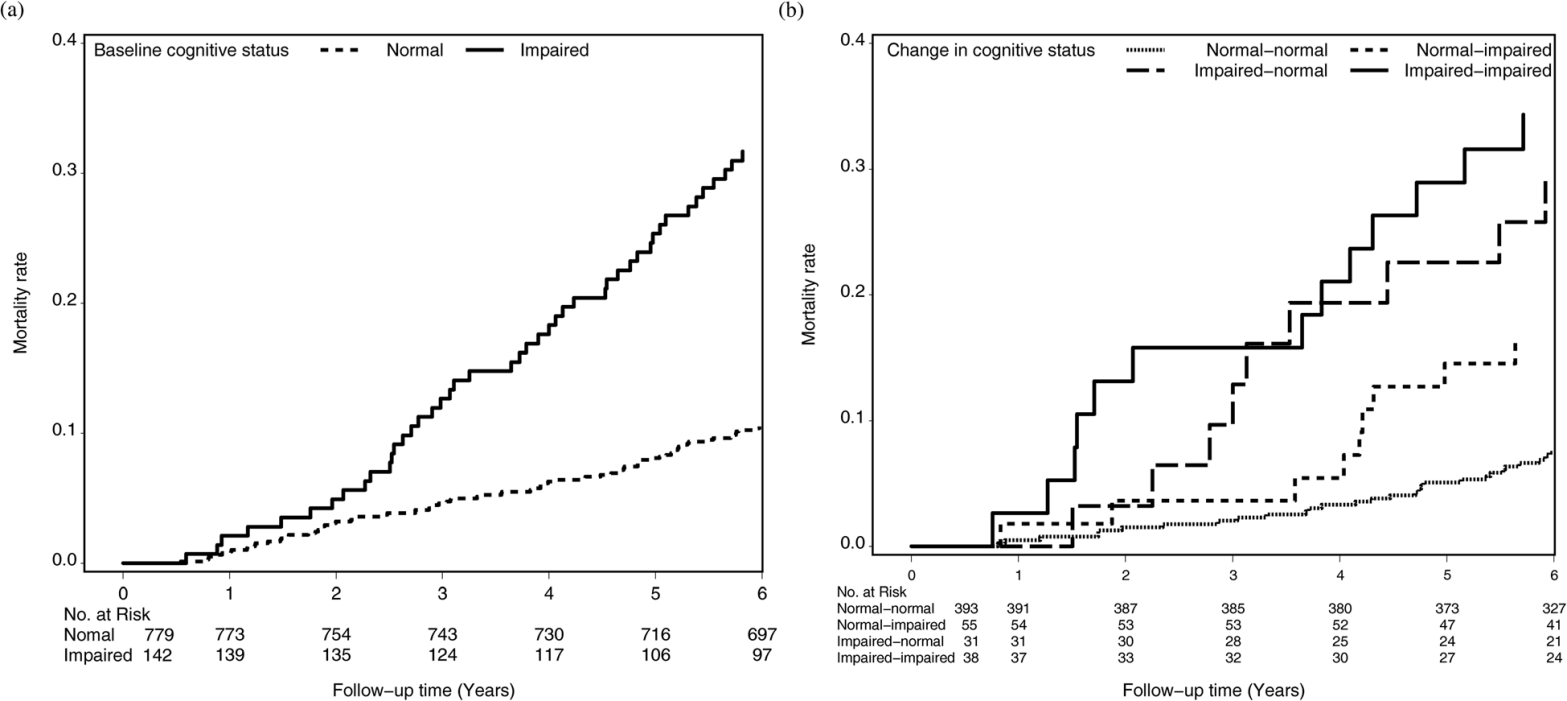
Survival curves of death for (**a**) baseline cognitive status and (**b**) one-year transition in cognitive status

**Fig. 3:**
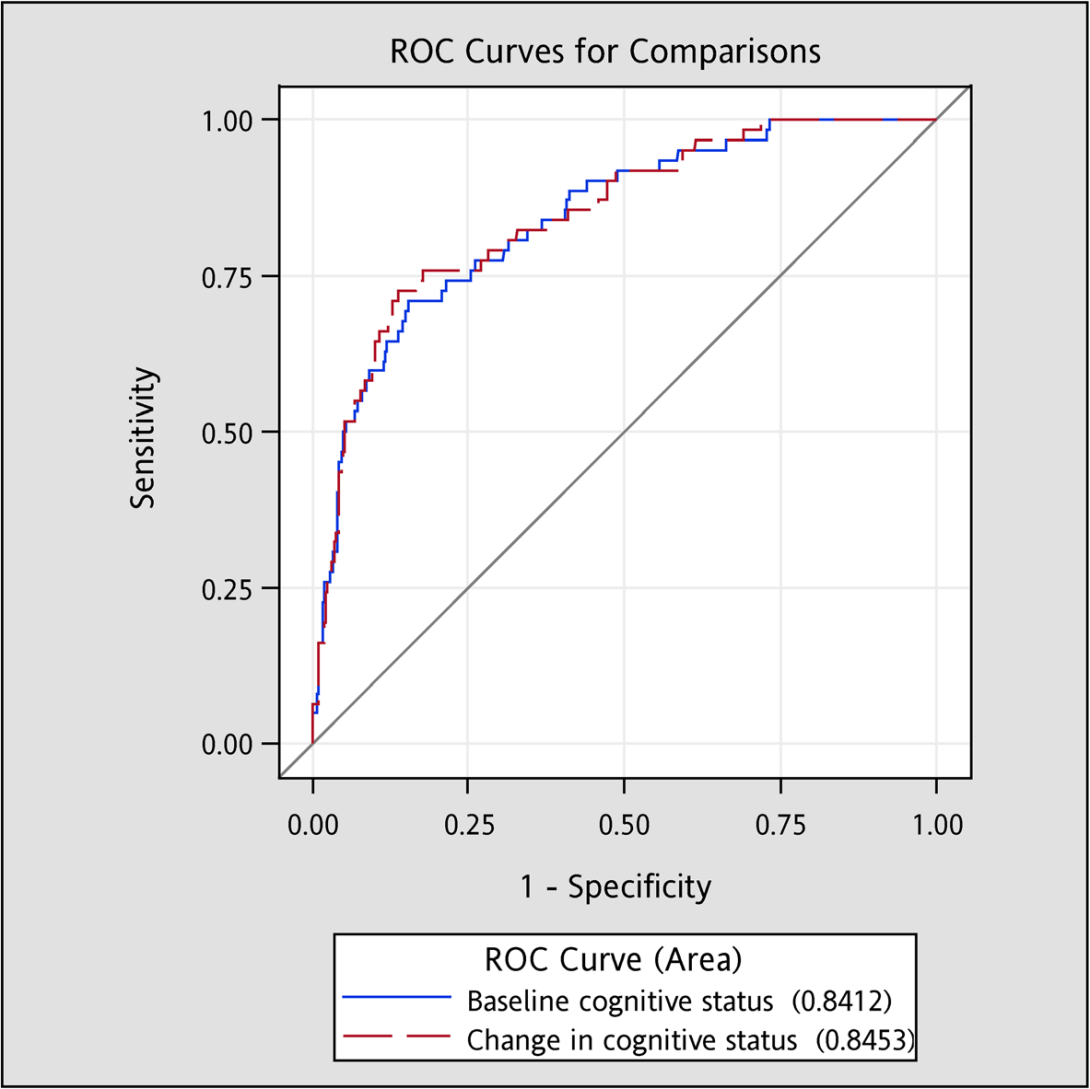
The areas under the receiver operating characteristic curves for all-cause mortality: baseline cognitive status versus one-year transition in cognitive status

The Kaplan–Meier estimates are plotted in Fig. 2. Participants who were cognitively impaired at baseline, reversed from impaired to normal, and remained impaired were at a high risk of death. The results were compatible with the above Cox modeling and served as a supplement for visual illustration. The AUCs in Fig. 3 demonstrate that the baseline cognitive status and one-year change in the cognitive status had no differential predictive ability for all-cause mortality (C-statistic: 0.8412 and 0.8453, respectively, *p* value: 0.55).

**Fig. 4:**
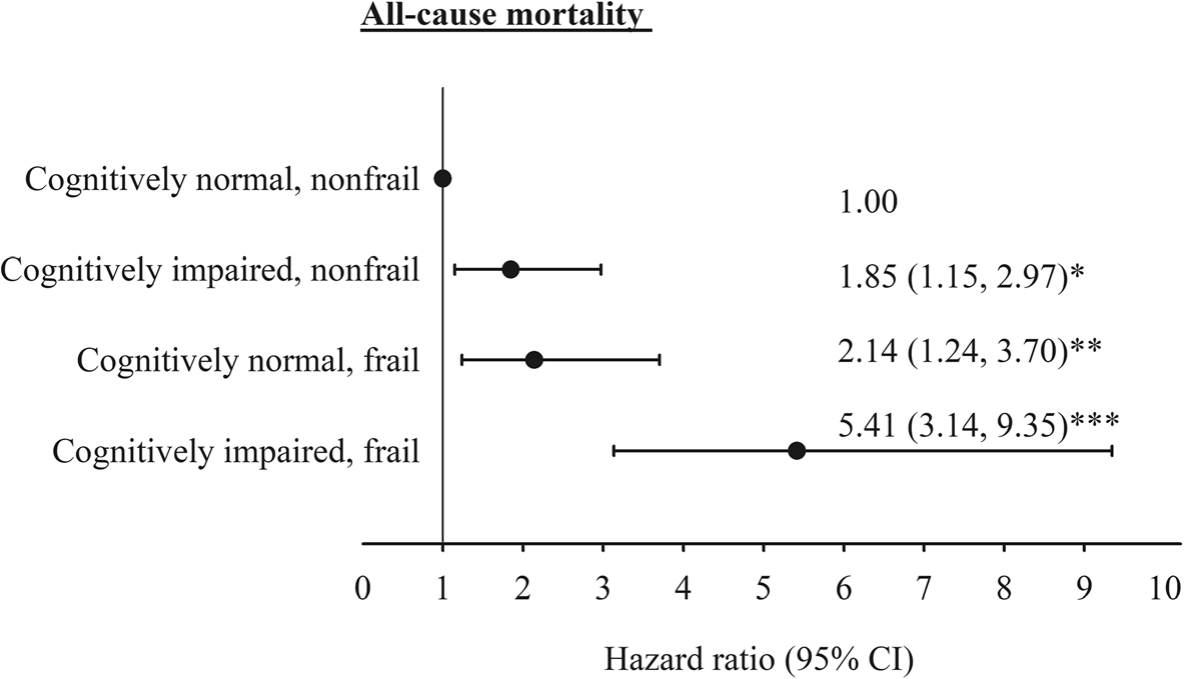
The multivariate adjusted hazard ratios of mortality for baseline cognitive impairment and frailty. Multivariate adjustment for age, sex, education, marital status, BMI, smoking, alcohol drinking, physical activity, exercising program, hypertension, diabetes mellitus, heart disease, hyperlipidemia, gout, hyperuricemia, arthritis, osteoporosis, stroke, cataract, cancer, fall history, and sleep disturbance. *: *p* < 0.05; **: *p* < 0.01; ***: *p* < 0.001

We further stratified the participants into two-by-two combinations of baseline cognitive function and frailty status. Compared with the elders who were cognitively normal and nonfrail at baseline, the HR (95% CI) for those cognitively impaired but nonfrail, cognitively normal but frail, and cognitively impaired and frail were 1.85 (1.15, 2.97), 2.14 (1.24, 3.70), and 5.41 (3.14, 9.35), respectively. Although cognitive impairment and frailty were independent mortality risk factors, we showed an additive effect and a slightly stronger association of frailty than cognitively impairment with all-cause mortality (Fig. 4).

## Discussion

This study demonstrates that baseline cognitive function predicts all-cause mortality in a six-year follow-up of urban community-dwelling older adults in Taiwan. Compared with older adults with normal cognition, those with impaired cognition at baseline were at a 2.08-fold risk of death after multivariate analysis of the fully adjusted model. Compared with older adults who remained cognitively normal over 1 year, those who reversed from cognitively impaired to normal had a 2.87-fold risk of death, whereas those who persisted to be cognitively impaired had a 3.79-fold increased risk of death. Older adults who remained cognitively impaired through the first year were the most risky subgroup. Those who reverted from impaired to normal cognition were still at a significantly higher risk of death, while those who converted from normal to impaired cognition were not, in the fully adjusted multivariate model. The fact implies that baseline cognitive impairment matters rather than cognitive progression during one-year period. This result is opposed to a previous study showing an increased risk of mortality primarily in elders with recent cognitive decline [11]. One explanation of the inconsistency is that the impact of cognitive impairment on survival may take longer than 5 to 6 years to develop in our cohort. Furthermore, we do not have a complete trajectory of cognitive function throughout the follow-up time. As early cognitive impairment is a reversible transition state, we are not sure if those who converted from normal to impaired cognition in the first year will progress toward the same direction or reverse to a better cognitive status. One may argue to repeat cross-sectional screening of the MMSE within shorter time intervals to account for continuity of cognitive change, but there potentially exists learning effect between the closely repeated examinations of a participant, resulting an overestimation of the latter MMSE scores. In such case, some of those who reverted from impaired to normal cognition may be indeed persistently impaired ones. Thus, it is impractical to refute the importance of any cognitive transition beyond 1 year, but our study would rather emphasize the predictive value of persistency of cognitive impairment during the first year.

Our findings that cognitive function predicts mortality are consistent with previous studies. Two European studies found 1.5 to 1.8-fold higher risk of mortality for incident dementia patients over an intermediate follow-up period [33, 34]. An inverse and dose-response relationship between cognitive function and mortality had been noted in demented [35] and non-demented elders [36]. A Korean cohort study showed a 1.59-fold increased risk of death for mild cognitive impairment patients compared with cognitively normal controls, and the population attributable risk of death due to mild cognitive impairment increased with age [37]. Although these results are consistent, the magnitudes differ due to several reasons, including different populations studied, non-uniform definitions of cognitive impairment, and varied lengths of follow-up.

The main outcome of our study is the all-cause mortality. This choice is reasonable in that a large proportion of older people have comorbidities and death may be attributed to multiple causes. Cardiovascular diseases are the leading cause of death among the elderly population worldwide. A Chinese study over 20 years of follow-up revealed baseline cognitive impairment assessed by the MMSE was associated with cardiovascular mortality in the competing risk model which accounted for the competing event of non-cardiovascular deaths [38]. The relationship between cognitive impairment and cardiovascular diseases is complex. Even subtle cognitive impairment reduces capability of performing tasks and activities of daily living in patients with cardiovascular diseases and risk factors [39]. This may seriously affect their self-care ability, consequently increasing the risk of cardiovascular deaths. On the other hand, cardiovascular diseases accelerate cognitive decline, especially in the elderly [40]. Aside from vascular dementia attributed to stroke, patients with cardiovascular diseases other than stroke still have a higher risk of senile dementia [41]. In fact, the actual mechanism of how cognitive impairment predicts future all-cause mortality is even more complex. A preventive strategy for some causes of death may be a risk factor for others. Nonetheless, previous studies revealed that mortality risks from cerebrovascular and respiratory diseases are particularly higher among older people with mild cognitive impairment [37] and dementia [34] than those without.

In our study, we classified individuals’ cognitive function into normal or impaired in terms of their MMSE scores, instead of clinicians’ diagnosis of dementia or mild cognitive impairment. We did not distinguish different subtypes of dementia, but one study showed that survival is similar in Alzheimer’s disease and vascular dementia [35]. The validity of MMSE had been challenged in assessment of cognition in patients with late-life depression, for a large percentage of cognitively impaired depressed elders would be misclassified as cognitively normal [42]. Meanwhile, the authors showed a substantial improvement of the sensitivity of the MMSE in this subgroup by raising the cutoff score from 24 to 27, just as what we set for those receiving formal education of more than 6 years.

In our previous work, we had demonstrated that frailty is a strong predictor for all-cause mortality [26]. In the current study, we investigated the role of cognitive impairment–frailty interaction in the risk of mortality. We showed that older people who were frail and cognitively impaired had the highest risk of death. Those who were frail but cognitively normal had a slightly greater hazard ratio for death than those who were cognitively impaired but not frail. This magnitude was stronger than that in a 4-year study in China, which showed a 2.13-fold increased mortality risk for older people with combined frailty and cognitive impairment but not for those with frailty alone or cognitively impairment alone [43]. The minor difference may be due to the different definitions of frailty in the two studies. The China study adopted Rockwood frailty index rather than Fried’s frailty phenotype.

The present study is one of the scarce numbers of studies that investigate cognitive transition in relation to mortality [11, 24]. As the educational levels vary to a great extent in Taiwanese population, one of the strengths of our study is to adapt the MMSE scores to educational attainment of an individual. Our study was conducted among a representative sample of community-dwelling older adults, rather than in institutions or clinical settings, thus the results can be applied to the general population. We also set up standardized protocols and instruments, a strict personnel training process, and vigorous quality assurance programs. However, some limitations exist. Our sample represented a Taiwanese metropolitan older population, thus, the findings may not be generalizable to older adults residing in rural areas. We excluded residents in the nursing home, who were at a high risk of cognitive impairment and death. Hence, we may have underestimated their association. The non-participation rate was high, indicating that potential selection bias may exist, although similar distributions of age and gender for the sample and Taichung population were found. Finally, cognitive transition was defined by the MMSE of the participants measured at a fixed time interval, thus we were unable to determine the exact time point of transition. As in many other cohort studies, the analysis results can be biased if statistical analysis does not take into account the problem of interval censoring [34]. As cognitive transition is a continuous process and even reversible in its early phase, future investigation would be of greater value in regard to refinement of cognitive trajectories.

## Conclusions

In conclusion, baseline cognitive impairment was associated with increased all-cause mortality in Taiwanese community-dwelling individuals aged 65 years and above, and older adults remaining cognitively impaired over one-year period had an even higher risk of death. Besides, the association between cognitive impairment and mortality was modified by frailty. The elders who were both cognitively impaired and physically frail had the highest risk of mortality. These results are important in identifying high-risk elderly population and providing preventive strategies that might reduce mortality in older adults.

## Data Availability

The datasets generated and/or analyzed during the current study are not publicly available due to the policy declared by Ministry of Health and Welfare in Taiwan but are available from the corresponding author on reasonable request.

## Abbreviations

TCHS-E: Taichung Community Health Study for Elders
HRC: Human Research Committee
BMI: Body mass index
MMSE: Mini-Mental State Examination
HR(s): Hazard ratio(s)
CI(s): Confidence interval(s)
AUCs: Areas under the receiver operating characteristic curves
